# Anthelmintic Activity and Pathophysiological Effect of *Allium sativum* Extract–Based Copper Nanoparticles Against the Carcinogenic Liver Fluke, *Opisthorchis viverrini*

**DOI:** 10.1155/japr/7058749

**Published:** 2025-08-13

**Authors:** Patpicha Arunsan, Phornphitcha Pechdee, Sirichai Phinsiri, Alisa Boonsuya, Chutharat Thanchonnang, Nav La, Nattawut Keeratibharat, Nathkapach Kaewpitoon Rattanapitoon, Schawanya Kaewpitoon Rattanapitoon

**Affiliations:** ^1^Parasitic Disease Research Center, Suranaree University of Technology, Nakhon Ratchasima, Thailand; ^2^Institution of Research and Development, Suranaree University of Technology, Nakhon Ratchasima, Thailand; ^3^Faculty of Medicine, Vongchavalitkul University, Nakhon Ratchasima, Thailand; ^4^Public Health and Allied Health Sciences, Sirindhorn College of Public Health Suphanburi, Praboromarajchanok Institute, Suphanburi, Thailand; ^5^International University, Phnom Penh, Cambodia; ^6^School of Surgery, Institute of Medicine, Suranaree University of Technology, Nakhon Ratchasima, Thailand; ^7^FMC Medical Center, Nakhon Ratchasima, Thailand; ^8^Department of Family Medicine and Community Medicine, Institute of Medicine, Suranaree University of Technology, Nakhon Ratchasima, Thailand

**Keywords:** *Allium sativum*, anthelmintic activity, copper nanoparticles, *Opisthorchis viverrini*, pathophysiological effect

## Abstract

*Opisthorchis viverrini* has a significant role in cholangiocarcinoma (CCA), a leading cause of death in northeastern Thailand. Although praziquantel (PZQ) remains the standard treatment for *O. viverrini* infections, its use is associated with adverse side effects, and prolonged administration may increase the risk of CCA. In response, the Thai government has been actively promoting research into alternative treatments, including the use of medicinal plants. *Allium sativum* Lin. (garlic) has demonstrated potent anthelmintic effects against *O. viverrini* adult worms in earlier studies, suggesting its potential as an alternative treatment for opisthorchiasis. Therefore, this study aims to investigate the potential of garlic–copper nanoparticles (CuNPs) (G-CuNPs) as a novel therapeutic approach for *O. viverrini* infections by enhancing the delivery efficiency of bioactive compounds in garlic. G-CuNPs were synthesized by the ionic gelation method and characterized using Fourier transform infrared (FTIR) spectroscopy, gas chromatography–mass spectrometry (GC-MS), and scanning electron microscopy (SEM). Furthermore, the antiparasitic efficacy of G-CuNPs against *O. viverrini* adult worms was evaluated through *in vitro* assays (conducted in triplicate), including assessments of motility and viability rates, the tegumental alteration using SEM analysis, and reactive oxygen species (ROS) production. The result of FTIR analysis confirmed stable interactions between garlic extract and CuNPs, while GC-MS identified disulfide derivatives with anti-inflammatory properties as the primary compounds. SEM revealed spherical nanoparticles approximately 226.2 nm in size, suitable for biological applications. Moreover, G-CuNPs effectively inhibited *O. viverrini* adult worms' motility and caused tegumental damage to the parasites, likely due to increased ROS levels. According to these results, G-CuNPs demonstrate significant potential as an alternative treatment for liver fluke infections. However, additional studies are necessary to thoroughly assess their safety, optimize drug delivery mechanisms, and explore their broader clinical applications.

## 1. Introduction

The current situation regarding parasitic infections among the Thai population has shown a continuous upward trend. Thailand's tropical and humid environment, which favors infection and dissemination, is the main cause of this. Contributing factors include long-standing local practices, such as regional dietary customs, particularly the consumption of partially cooked food, a major contributor to gastrointestinal parasitic infections [1]. According to the Department of Disease Control, the national parasitic infection rate is 9.79%, with the southern region reporting the highest rate at 13.43%, followed by the northeastern region at 11.86%, and the northern and central regions at 9.68% and 6.03%, respectively. Hookworm is the most common helminth infection (4.47%), followed by *Opisthorchis viverrini* (2.2%), which is particularly endemic in the northeast [2]. According to the Ministry of Public Health's statistics, *O. viverrini* infection is a major risk factor for liver and bile duct malignancies, which are Thailand's top causes of death. *O. viverrini* is one of just 10 infectious agents that are directly carcinogenic to humans, having been classified as a class one carcinogen by the International Agency for Research on Cancer (IARC) since 1994, with chronic infection strongly associated with cholangiocarcinoma, particularly in northeastern Thailand [3]. The highest incidence of bile duct cancer is found in the northeastern region, where patients often succumb rapidly after diagnosis, leading to substantial economic loss for the country due to healthcare costs. Currently, praziquantel (PZQ) is used to treat *O. viverrini* infections. However, this drug has important adverse effects [4]. Moreover, prolonged use of PZQ may increase the risk of abnormal bile duct development, potentially leading to bile duct cancer [4–7]. Currently, the government has focused on promoting public education and research initiatives aimed at leveraging domestic resources for sustainable solutions, with a particular emphasis on Thai medicinal herbs.

*Allium sativum* Lin. (garlic), a common household herb, has been extensively studied for its health benefits, including immune-boosting properties and antimicrobial activity [8]. Key bioactive compounds in garlic, such as allicin, organic sulfides, saponins, phenolic compounds, and polysaccharides, have demonstrated significant antiparasitic properties [9]. Garlic has demonstrated promising anthelmintic activity against several trematode species. Studies have shown that garlic extract significantly reduces worm burden and tissue pathology in mice infected with *Echinostoma caproni* [10] and schistosomiasis [11–13]. Specifically, garlic extract has shown in vitro efficacy against *O. viverrini*, a liver fluke endemic in Southeast Asia, by disrupting the parasite's tegument and morphology [14]. These findings highlight garlic's potential as a natural therapeutic agent against trematode infections and support further investigation into its mechanisms and applications in endemic areas. Studies have shown that garlic extracts can disrupt calcium ion exchange in the cell membranes of parasites, impairing mitochondrial function and energy production within parasitic cells [15]. Garlic has been effective in both larval and adult stages of nematodes such as *Haemonchus contortus* by reducing the parasitic infection rate in mice [16]. Additionally, crude garlic extract has been found to reduce parasitic infections in mice infected with *Anisakis* species when administered both before and after infection [17]. Electron microscopy studies revealed morphological changes, such as swelling, in intestinal flukes treated with garlic extract [10], highlighting the potential of garlic as an antioxidant and immunomodulator in parasitic infections [11, 18]. Also, garlic oil extract has been reported to paralyze and inhibit the movement of *Fasciola gigantica*, a large liver fluke affecting both humans and animals [19]. Especially, previous research has unequivocally affirmed the anthelmintic effectiveness of garlic extract against *O. viverrini*, consistent with earlier reports. The study highlights the efficacy of garlic extract in targeting *O. viverrini* adult worms [14].

Given the side effects of current anti-parasitic treatments, there is a need to explore more effective and safer alternatives. Nanotechnology, which has been successfully applied in antibacterial, antifungal, and antiviral treatments, as well as in biosensing, offers unique properties, such as improved solubility and specificity, that enhance the efficacy of herbal extracts [20, 21]. The green synthesis of herbal nanoparticles is simple, environmentally friendly, and involves the use of polyphenolic compounds, such as flavonoids, terpenes, alkaloids, and tannins, which have demonstrated anti-parasitic and antiprotozoal activities [22]. These compounds can act as reducing agents in the synthesis of metal nanoparticles, such as silver, gold, or copper, enhancing the stability and bioactivity of the herbal extract [23]. Copper nanoparticles (CuNPs) are preferred for their strong ROS generation, which enhances antimicrobial and cytotoxic effects, and their cost-effectiveness compared to silver and gold. While biodegradable nanoparticles like lipid or chitosan-based types are safer, they lack CuNPs' potent ROS activity. However, CuNPs' potential toxicity necessitates careful evaluation [24, 25]. CuNPs are recognized as safe, nontoxic inorganic materials with strong antibacterial and antifungal properties [26]. Consequently, this study is aimed at exploring a garlic-based copper nanoparticle (G-CuNPs) formulation through biogenic synthesis, targeting the adult stages of *O. viverrini*, as a potential target for alternative treatments that enhance the efficiency of drug delivery in the treatment of parasitic infections in both humans and animals.

## 2. Materials and Methods

### 2.1. Ethics Statement

This study complied with biosafety regulations and was approved by the Biosafety Control Committee at Suranaree University of Technology. This study was conducted in accordance with Biosafety Level 2 (BSL-2) guidelines and approved by the Biosafety Control Committee of Suranaree University of Technology (Project code: IBC-65-09; Certification: SUT-IBC-009/2022). Furthermore, ethical approval for the use of animals was obtained from the Institutional Animal Care and Use Committee (IACUC) at Suranaree University of Technology, under Certification Number SUT-IACUC-0022/2022 and animal use Permit Number U1-01369-2015.

### 2.2. Crude Garlic Extraction Procedure

The garlic cloves were purchased from a local market in Nakhon Ratchasima province. They were weighed at 150 g for extraction, peeled, thoroughly washed with distilled water (DW) and finely ground using a mechanical grinder. The resulting garlic juice was squeezed through a filtering four-layered muslin cloth into a sterile beaker. Subsequently, the extract solution was centrifuged at 4500 rpm for 20 min to separate the precipitate. The supernatant was filtered using Whatman filter paper No. 1 (GE healthcare life sciences, Ohio, United States) [14]. The garlic crude extract was stored at 4°C for further use in the synthesis procedure.

### 2.3. Synthesis of Garlic Crude Extract Coated CuNPs

A 0.01 M copper nitrate (Cu(NO_3_)_2_) [27] (Sigma-Aldrich, Missouri, United States) solution was prepared following the basic chemical formula:

(1) $$\begin{array}{c} \frac{W}{M}=\frac{\text{VC}}{1,000}, \end{array}$$

where V is the desired volume of the solution, C is the desired concentration, W is the weight of the Cu(NO_3_)_2_, M is the molecular weight of Cu(NO_3_)_2_ = 241.60 g/M.

Substitute the following values: *W*/241.60 g/*M* = 200 mlx0.01 M/1000.

 $$\begin{array}{c} W=0.482\text{ g}/\text{M}. \end{array}$$

Weigh 0.482 g of Cu(NO_3_)_2_ and dissolve it in 200 mL of deionized (DI) water. Garlic crude extract 10 mL was added to 80 mL of the 0.01 M Cu(NO_3_)_2_ solution. Then, 10 mL of hydrazine hydrate (Sigma-Aldrich, Missouri, United States) was introduced to the mixture [27]. The mixture was stirred continuously on a magnetic hotplate stirrer at room temperature under ambient light for 24 h. The resulting product was centrifuged at 3000 rpm for 20 min to collect the precipitate. The precipitate was washed three times with DI water and centrifuged at 3000 rpm. Finally, the precipitate was dried at 50°C for 5 h and ground into a fine powder for subsequent testing.

### 2.4. Physical Characterizations of G-CuNPs

Fourier transform infrared (FTIR) spectroscopy analysis was conducted on the chemical composition to identify the types of biomolecules in G-CuNPs, in comparison with Cu(NO_3_)_2_ and garlic crude extract. The procedure involved placing the samples of all three substances into the sample compartment of the FTIR spectrometer, followed by measurement in the wavelength range. For the analysis, approximately 10 mg of each substance was placed in the sample holder of the Vertex 70 FTIR spectrometer (Bruker, Fällanden, Switzerland), and measurements were performed over the wavelength range of 400–600 cm^−1^.

Gas chromatography–mass spectrometry (GC-MS) was utilized for bioactive compounds analysis to combine the principles of GC-MS for compound analysis. The Agilent 7890A gas chromatograph (Agilent, California, Untied States) was used to determine the types and quantities of compounds in 1 g/mL of each sample through injection into a chromatographic column. The separated compounds were subsequently detected and quantified based on their mass-to-charge ratios (m/z) using the Agilent 7890B mass spectrometer (Agilent, California, USA). The resulting data were represented as mass spectra, which were compared against a database to identify compounds and evaluate their likelihood of presence, thereby eliminating the requirement for standard substances for comparison.

The morphological surface of G-CuNPs was visualized using scanning electron microscopy (SEM). G-CuNP powder was applied onto carbon tape to form a thin layer, ensuring minimal thickness. The sample was then coated with gold using a sputtering device (Leica CPD 300, Vienna, Austria) to enhance conductivity. Imaging was conducted using a field emission scanning electron microscope (FESEM) (Carl Zeiss Auriga, Dresden, Germany) at an accelerating voltage of 3.0 kV. The particle size of the G-CuNPs was measured using ImageJ software (https://imagej.net/ij/download.html).

### 2.5. Anthelmintic Activity of G-CuNPs

*O. viverrini* metacercariae were isolated from naturally infected cyprinid fish collected in an endemic region encompassing Nakhon Ratchasima and Chaiyaphum Provinces, Northeastern Thailand. Fresh cyprinid fish were digested in a 0.25% pepsin–hydrochloric acid solution and incubated at 37°C for 2 h. The resulting solution was filtered using a 0.85% normal saline solution in a sedimentation jar. Metacercariae were identified based on their morphology by a parasitologist using a stereomicroscope [28]. Male Syrian golden hamsters (aged 8 weeks) were infected orally with 50 *O. viverrini* metacercariae through intragastric intubation. Over 3 months, the development of *O. viverrini* adult worms occurred in the liver bile ducts of the hamsters. After this period, the infected hamsters were sacrificed, and adult worms were collected from the liver bile ducts for use in experimental studies [14, 29].

*O. viverrini* adult worms were divided into six groups for exposure to various substances (5 worms/group × 3 replicates). This approach aligns with WHO guidelines, which allow smaller sample sizes in early-phase assays depending on the parasite species and study objective [30]. Group 1: negative control group, maintained in Roswell Park Memorial Institute (RPMI) 1640 culture medium. Group 2: positive control group, treated with standard PZQ (HK Pharmaceutical, Bangkok, Thailand) at a concentration of 20 mg/mL dissolved in RPMI-1640 culture medium [14]. Group 3: treated with a 100 *μ*M Cu(NO_3_)_2_ solution. Groups 4–6: treated with G-CuNPs at concentrations of 50, 100, and 150 ppm, respectively. Each concentration was supplemented with 100 *μ*g/mL of penicillin–streptomycin (Pen-Strep) antibiotic (Cytiva, Pascing, Austria) to prevent bacterial contamination during the process. After that, all groups were exposed at different time intervals (0, 5, and 30 min; 1, 3, 6, 12, and 24 h) under 37°C atmosphere.

The motility assessment of *O. viverrini* adult worms was assessed by examining adult worms under a stereomicroscope and assigning scores based on established criteria (3 = *full-body movement*, 2 = *partial-body movement*, 1 = *immobile but alive*, and 0 = *dead*) as outlined in previous studies [31, 32]. The relative motility (RM) value was calculated using motility scores from all experimental groups. In the negative control group, where all parasites were assigned a score of 3, the RM value was 100. A reduction in RM observed in the G-CuNPs group indicated stronger inhibition of motility. The RM value was determined using the formula provided below [33, 34].

(2) $$\begin{array}{c} \text{Motility index }\left(\text{MI}\right)=\frac{\sum nN}{\text{N}}, \end{array}$$

(3) $$\begin{array}{c} \%\text{Relative motility }\left(\text{RM}\right)\text{value}=\frac{\text{MI test}\times 100}{\text{MI control}}, \end{array}$$

where *n* is the motility score and *N* is the number of worms with the score of “*n*”.

Furthermore, the survival index (SI) was calculated to assess the percentage of live adult worms after various incubation times. Adult worms were classified as dead if they exhibited a motility score of 0, while those with scores of 1, 2, or 3 were considered alive. The SI was determined using the formula below [33, 34]:

(4) $$\begin{array}{c} \%\text{Survival index }\left(\text{SI}\right)=\frac{\text{Number of live adult worm }\left(\text{each group}\right)}{\text{Total adult worm }\left(\text{each group}\right)}\times 100. \end{array}$$

The viability of *O. viverrini* adult worms was observed after 24 h of exposure. Adult parasites were stained with a 0.4% Trypan blue stain (Thermo Fisher Scientific, Massachusetts, United States) at room temperature for 3 min. Then, the adult worms were destained three times with 1x phosphate buffer saline (PBS), and the viability of parasites was evaluated under a light microscope [14, 29]. Live adult worms were identified by the absence of staining, while dead parasites were marked by blue staining from Trypan blue, indicating cell death [29].

### 2.6. Pathophysiological Effect of G-CuNPs

SEM analysis was conducted 12 h postexposure. Adult worms were fixed in glutaraldehyde overnight at 4°C, washed in DW, postfixed with 1% osmium tetroxide (0.1 M PBS, pH 7.2) for 1 h, and dehydrated through graded acetone. Samples were dried (Leica CPD 300), sputter-coated with gold, and examined via FESEM (Carl Zeiss Auriga) at 3.00 kV [14, 29].

The stress generation due to reactive oxygen species (ROS) was induced by G-CuNPs, triggering a cellular response in *O. viverrini* adult worms. It was measured after 6 h of incubation to establish a consistency between antioxidant defenses and ROS or free radicals. Adult worms were thoroughly rinsed with DW and subsequently exposed to a 30 *μ*M solution of the fluorogenic dye 2⁣′,7⁣′-dichlorodihydrofluorescein diacetate (H2DCFDA) (Med Chem Express, New Jersey, United States). The worms were incubated in darkness at 37°C for 30 min to facilitate dye uptake. Following incubation, the samples were washed with DW to remove any excess dye. Fluorescence imaging was performed using an inverted fluorescence microscope with excitation and emission wavelengths of 488 and 525 nm, respectively (Nikon, Tokyo, Japan) [14, 29]. ROS levels were quantified by analyzing fluorescence microscopy images using ImageJ software. The images were identified and selected using the drawing/selection tools. Subsequently, the image menu was accessed to adjust color settings and split the channels for individual analysis. Measurement parameters were configured by navigating to the “set measurements” option, where area, integrated density (IntDen), and mean grey value were selected for data quantification [35]. Also, the corrected total worm fluorescence (CTWF) was calculated by subtracting the IntDen from the product of the selected adult worm's area and the mean background fluorescence [35].

(5) $$\begin{array}{c} \text{CTWF}=\text{Integrated density}-\left(\text{Area of selected cell}\times \text{fluorescence of background reading}\right). \end{array}$$

### 2.7. Data Analysis

RM and SI values were calculated based on motility scores and presented as mean ± standard deviation (SD). One-way ANOVA followed by Tukey's HSD post hoc test was performed using IBM SPSS Statistics Version 26 (SPSS Inc., Chicago, United States). Statistically significant differences were considered when the probability value (*p* value) was < 0.05.

## 3. Results

### 3.1. Physical Characterizations of G-CuNPs

**Figure 1:**
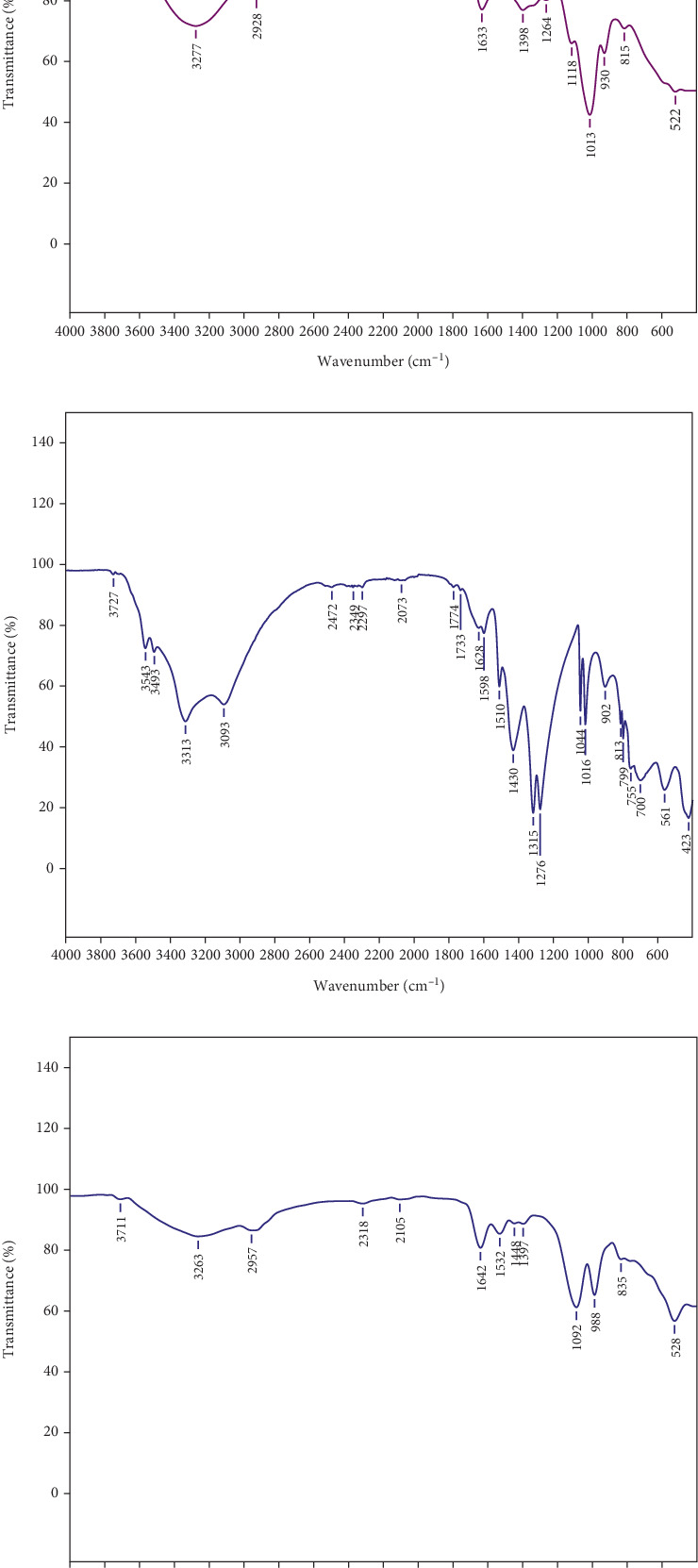
FTIR spectra identifying functional groups in (a) garlic crude extract, (b) Cu(NO₃)₂, and (c) G-CuNPs. The spectra illustrate the interaction between garlic compounds and copper ions, supporting nanoparticle stabilization.

FTIR spectroscopy revealed distinct functional groups contributing to the synthesis and stabilization of G-CuNPs. In the garlic crude extract spectrum (Figure 1a), prominent peaks at 3723 and 3277 cm^−1^ indicated hydroxyl (O-H) and hydrogen-bonded functionalities. The 2928 cm^−1^ peak represented aliphatic C-H stretching. Peaks at 1633, 1264, and 1118 cm^−1^ corresponded to carbonyl (C=O), esters/ethers, and polysaccharides, respectively. Sulfur-containing groups, including C-S and aromatic structures (815 and 522 cm^−1^), emphasized the extract's bioactivity. The Cu(NO₃)₂ spectrum (Figure 1b) displayed peaks at 3727, 3543, and 3493 cm^−1^ due to O-H vibrations from coordinated water. Peaks between 1774 and 1436 cm^−1^ were attributed to asymmetric and symmetric stretching of nitrate (NO₃^−^) ions. Cu–O bonds were confirmed by absorption at 700, 561, and 423 cm^−1^. In the G-CuNP spectrum (Figure 1c), characteristic peaks appeared at 3711 and 3263 cm^−1^ (O-H stretching), 1642 cm^−1^ (C=O from polyphenols), and 1092 cm^−1^ (alcohol/ether groups). A peak at 528 cm^−1^ confirmed Cu-O bond formation, providing direct evidence of successful capping and stabilization by garlic functional groups. These findings confirm the integration of garlic-derived biomolecules with CuNPs, enabling potential biological activity.

**Figure 2:**
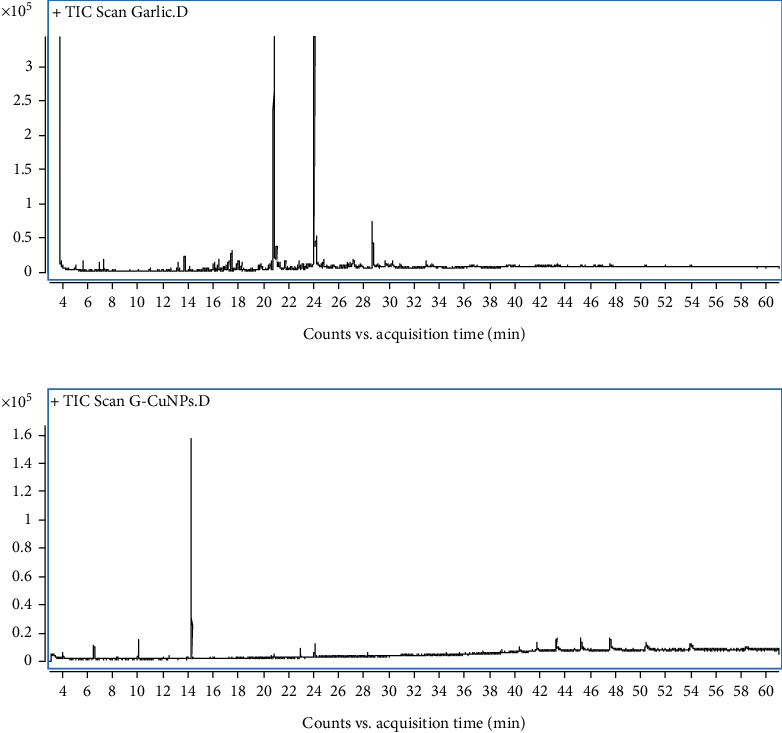
GC-MS chromatograms showing compound separation in (a) garlic extract and (b) G-CuNPs. Peak identification based on mass spectra indicates sulfur-containing compounds as major constituents, essential for bioactivity and nanoparticle stability.

GC-MS analysis revealed the chemical profiles of garlic extract and G-CuNPs (Figure 2). The garlic extract chromatogram (Figure 2a) showed 16 compounds, including major sulfur-based constituents: diallyl disulfide (0.41%), 3-vinyl-1,2-dithiacyclohex-5-ene (0.43%), 3-vinyl-1,2-dithiacyclohex-4-ene (0.19%), and 2-propenylthioacetonitrile (0.35%). Dodecyl acrylate (79.84%) was the dominant component, with 1-dodecanol (15.21%) and several hydrocarbons and esters also present (Table 1). In G-CuNPs (Figure 2b), nine compounds were identified, dominated by disulfide, dipropyl (71.87%). Other sulfur-containing compounds included disulfide, methyl propyl (7.76%) and benzothiazole, 2-(methylthio) (3.45%). Additional minor components such as benzoyl isothiocyanate (1.90%), sulfurous acid esters, and dodecyl acrylate (3.75%) contributed to nanoparticle bioactivity. Trace levels of limonene (0.74%) and ethyl hexadecanoate (0.98%) were also detected (Table 2). These results emphasize the sulfur-rich nature of the nanoparticles, consistent with enhanced bioactivity observed in subsequent assays. The GC-MS data were analyzed with GC-QQQ software, and compounds were identified using the NIST Mass Spectral Search 2.0 library. Error bars and retention indices have been included in corresponding tables. Refer to Tables 1 and 2 for compound details.

**Figure 3:**
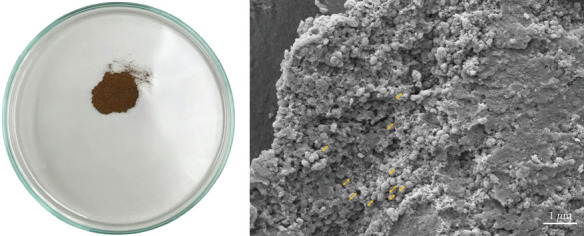
SEM characterization of G-CuNPs. (a) The synthesized G-CuNPs appeared as a dark brown powder. (b) SEM micrograph showed spherical nanoparticles with an average size of ~226.2 nm; yellow arrows indicate particle clusters. Scale bar = 1* μ*m.

G-CuNPs appeared as a fine dark brown powder (Figure 3a). SEM analysis revealed that the nanoparticles were predominantly spherical, with some aggregation. The average particle diameter was approximately 226.2 ± 25.37 nm (Figure 3b), indicating suitability for biomedical applications. The nanoscale size and morphology are consistent with enhanced cellular interactions and stability.

### 3.2. Anthelmintic Activity of G-CuNPs

**Figure 4:**
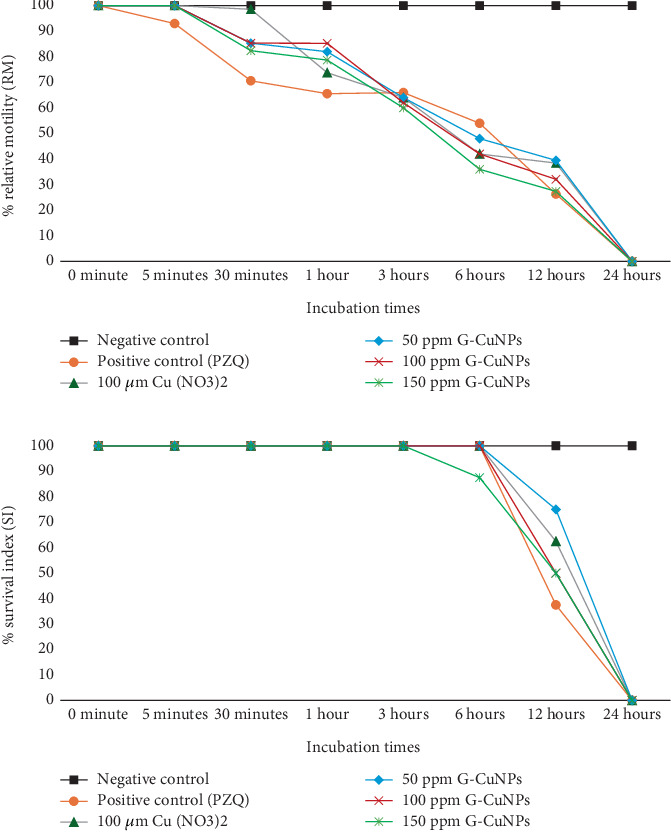
Inhibitory effects of G-CuNPs on adult *O. viverrini*. (a) RM and (b) SI values for each treatment group across time points. Complete loss of motility and viability was observed by 24 h in all treatment groups, excluding the negative control.

The anthelmintic effect of G-CuNPs was evaluated by measuring RM (Figure 4a) and SI (Figure 4b) at specified intervals. In the negative control group, RM and SI remained at 100% throughout the 24-h period. In contrast, PZQ treatment reduced RM to 70.59% at 30 min and to 26.32% at 12 h, with complete inhibition by 24 h (RM and SI = 0%). Cu(NO₃)₂ and G-CuNP treatments at all tested concentrations (50, 100, and 150 ppm) also showed time- and dose-dependent reductions in RM and SI. Notably, G-CuNPs at 150 ppm reduced RM to 36.00% at 6 h and 27.37% at 12 h, with complete inhibition (0% RM and SI) at 24 h. The same trend was observed in the 100 and 50 ppm groups, though at slower rates. These findings indicate that G-CuNPs effectively suppress worm motility and survival in a concentration-dependent manner.

**Figure 5:**
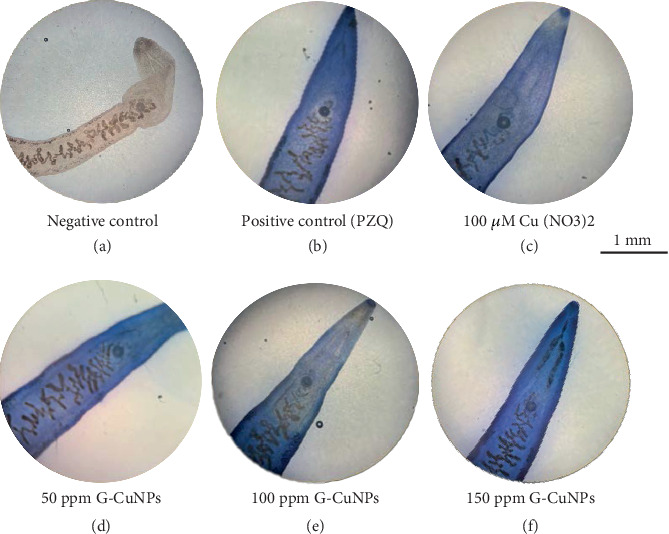
Trypan Blue staining of treated adult *O. viverrini*. (a) Negative control (unstained), (b) PZQ-treated, (c) Cu(NO₃)₂-treated, and G-CuNP-treated worms at (d) 50 ppm, (e) 100 ppm, and (f) 150 ppm. Blue staining indicates loss of viability. Scale bar = 1 mm.

Trypan Blue viability staining confirmed these findings. The G-CuNP group at 150 ppm exhibited the most intense blue staining, followed by 100 and 50 ppm, Cu(NO₃)₂, and PZQ groups, consistent with the severity of motility loss (Figure 5).

**Figure 6:**
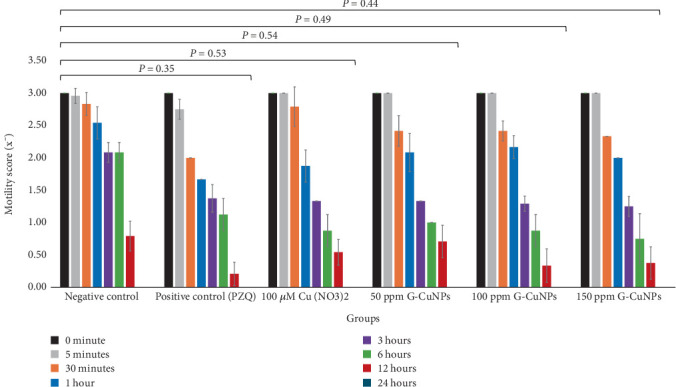
Comparison of average motility scores. Although treatment groups showed reduced motility, differences were not statistically significant (*p* > 0.05).

Statistical comparison using one-way ANOVA revealed no significant differences (*p* > 0.05) in average motility scores between the treatment and control groups (Figure 6). The lack of statistical significance may reflect biological variability and limited sample size (5 worms/group × 3 replicates).

### 3.3. Pathophysiological Effects of G-CuNPs

**Figure 7:**
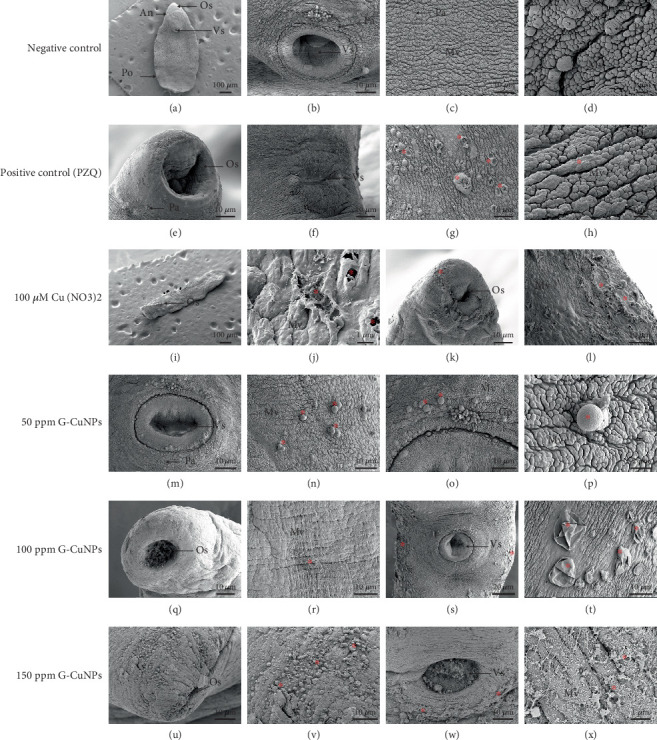
SEM images of tegumental alterations. (a–d) Control; (e–h) PZQ; (i–l) Cu(NO₃)₂; (m–p) G-CuNPs 50 ppm; (q–t) G-CuNPs 100 ppm; (u–x) G-CuNPs 150 ppm. Scale bars = 1, 2, 10, 20, and 100* μ*m. ⁣^∗^Swelling, desquamating, blistering, and deterioration of the tegumental surface. Abbreviations: An, anterior region; Po, posterior region; Os, oral sucker; Vs, ventral sucker; Pa, papillae; Mv, microvilli; Gp, genital pore.

SEM analysis of the worm tegument after 12-h exposure revealed dose-dependent morphological alterations. The negative control group exhibited intact tegumental structures, including oral and ventral suckers (Figures 7a, 7b, 7c, and 7d). In contrast, PZQ and Cu(NO₃)₂ treated worms displayed severe tegumental swelling, blistering, and desquamation (Figures 7e, 7f, 7g, 7h, 7i, 7j, 7k, and 7l). The severe tegumental swelling was evaluated using quantitative microscopic measurements, qualitative visual assessments, and comparative observations between the treatment and control groups. G-CuNPs at 50 ppm caused moderate tegumental damage with localized swelling and blister formation (Figures 7m, 7n, 7o, and 7p). At 100 and 150 ppm, worms exhibited pronounced degeneration, including extensive desquamation and tegumental peeling with exposure of subtegumental fibers (Figures 7q, 7r, 7s, 7t, 7u, 7v, 7w, and 7x).

**Figure 8:**
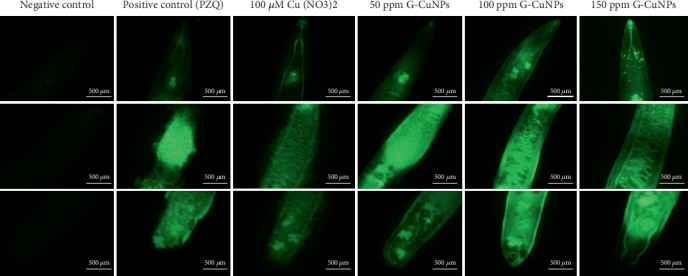
ROS expression in *O. viverrini* after 6-h treatment. Strong green fluorescence was observed in treated groups, indicating oxidative stress. Scale bar = 500* μ*m.

Fluorescence microscopy using H₂DCFDA dye revealed significant ROS accumulation in all treatment groups compared to the negative control. G-CuNPs treatment yielded the strongest green fluorescence, followed by PZQ and Cu(NO₃)₂ (Figure 8).

**Figure 9:**
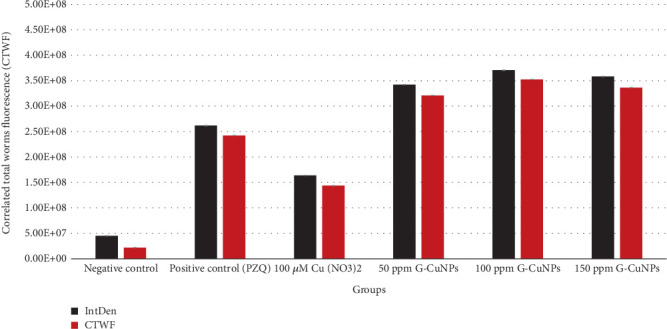
ROS quantification using ImageJ was carried out by analyzing IntDen and CTWF. IntDen represents the total fluorescence intensity, while CTWF accounts for background correction. Both values increased with rising concentrations of G-CuNPs, indicating enhanced oxidative stress. *O. viverrini* adult worms in the treated groups showed higher IntDen and CTWF levels compared to the negative control. The values increased sequentially in the order of 100 *μ*M Cu(NO_3_)_2_, PZQ, and G-CuNPs, respectively.

Quantitative fluorescence analysis confirmed elevated oxidative stress in treated worms. G-CuNPs (especially at 150 ppm) had the highest IntDen and CTWF values, indicating elevated ROS levels (Figure 9).

## 4. Discussion

Garlic contains active compounds such as organic sulfides, saponins, phenolics, and polysaccharides [9]. Among these, allicin, an organic sulfide, has been shown to possess antiparasitic properties [9, 35, 36]. Previous research demonstrated allicin's efficacy against various parasites including *Trypanosoma brucei*, *Trypanosoma congolense*, and *Trypanosoma vivax* [37], *Entamoeba histolytica* [38], *H. contortus* [7], *Anisakis* sp. [17], *Gyrodactylus turnbulli* [39], *Schistosoma mansoni* [40, 41], and *F. gigantica* [19]. Garlic also enhances immune response and reduces inflammation in infected animals, such as those infected with *Plasmodium yoelii* [42]. A recent study demonstrated that crude garlic extract exerted significant morphological damage and motility inhibition in *O. viverrini* adult worms, indicating its anthelmintic potential. Notably, that study did not employ nanoparticle formulations, whereas the present study introduces G-CuNPs, which enhance delivery and stability [14]. In this study, G-CuNPs significantly reduced worm motility and viability in a dose- and time-dependent manner, as evidenced by declining RM and SI values. Although statistical comparisons between treatment and control groups did not yield significant differences (*p* > 0.05), the biological trends were clear and consistent, supporting the efficacy of G-CuNPs. The lack of statistical significance may be attributed to small sample sizes, as highlighted by the WHO's guidelines on helminth efficacy testing [8, 30, 43]. The combined effect of garlic's bioactive compounds and CuNPs suggests a synergistic mechanism that impairs parasite metabolism and structural integrity while inducing oxidative stress. Compared to other herbal alternatives such as mangosteen rind, purple corn, ginger, betel nut, and *Thunbergia laurifolia*, G-CuNPs offer a nanoparticle-enhanced delivery platform with superior potential for clinical translation [44–49].

FTIR analysis confirmed the successful biosynthesis of G-CuNPs, with peaks indicating the presence of hydroxyl, amino, carbonyl, and Cu-O functional groups. These groups stabilize the nanoparticles by forming coordination bonds with Cu^2+^, preventing aggregation and enhancing bioactivity [50]. Similarly, GC-MS revealed the dominance of sulfur-containing compounds in both crude garlic extract and G-CuNPs. Notably, disulfide derivatives such as dipropyl disulfide and diallyl disulfide were prevalent, contributing to anti-inflammatory, antimicrobial, and antioxidant properties [51–53].

GC-MS analysis of garlic crude extract and G-CuNPs identified a variety of bioactive compounds with potential therapeutic relevance. The extract was predominantly composed of dodecyl acrylate (79.84%), known for its anti-inflammatory and antimicrobial effects [49], followed by 1-dodecanol (15.21%) with proven antimicrobial activity [54], and diallyl disulfide (0.41%), a sulfur-based compound recognized for its antioxidant, antimicrobial, and anticancer properties [55, 56]. Minor components such as limonene, isothiocyanates, and glycolaldehyde derivatives further supported antioxidant and anti-inflammatory activity [55]. The sulfur-rich composition of garlic extract appears to enhance the biological activity of G-CuNPs, particularly their antimicrobial, antioxidant, and anticancer properties, warranting further investigation into their underlying mechanisms and biomedical potential. The nanoparticle ultrastructure, with a mean diameter of ~226.2 nm and spherical morphology, is ideal for biological applications such as drug delivery. The tendency to form aggregates, likely due to van der Waals or electrostatic forces, suggests that future studies should address dispersion stability, particularly for therapeutic use [57]. CuNPs synthesized with garlic or ginger extracts have shown strong antibacterial, antifungal, antioxidant, and larvicidal activities [58–60]. These studies highlight the potential of plant-based metal nanoparticles for broad-spectrum antimicrobial and pest control applications [27, 61]. Anthelmintic activity was confirmed through Trypan Blue viability staining and SEM imaging. Trypan Blue distinguished live from dead worms, reinforcing RM and SI findings [29].

SEM revealed severe tegumental damage, swelling, blistering, and desquamation, especially at higher G-CuNP concentrations, surpassing the damage observed with PZQ. These findings align with prior observations in *S. mansoni*, *F. gigantica*, and *O. viverrini* exposed to garlic extract, supporting garlic's ability to compromise tegument integrity [14, 19, 40, 41].

While ROS play essential roles in processes such as signaling and immune responses, their effects can be both beneficial and detrimental depending on the context [62, 63]. In this study, G-CuNPs at concentrations of 50, 100, and 150 ppm induced elevated ROS levels in *O. viverrini* adult worms after 6 h of exposure, exceeding those observed in both positive (Cu(NO₃)₂) and negative control groups. This ROS generation indicates stress within the parasites, a typical response where increased ROS leads to cellular damage, disruption of respiration, and ultimately cell death caused by hypoxia and metabolic disturbances. The resulting reduction in ATP production triggers anaerobic metabolism, causing lactate buildup, decreased pH, lysosomal swelling, and enzyme release, which culminate in autolysis and tissue destruction [64]. These findings correspond with previous research on *H. contortus* treated with *Lansium parasiticum* aqueous extract-protected silver nanoparticles, which demonstrated a metabolic shift in response to ROS-induced oxidative stress [65]. Overall, the generation of ROS reflects oxidative stress within parasites, leading to cellular dysfunction and potential death [62, 63, 66]. These findings support previous research demonstrating the potential of green-synthesized CuNPs using garlic extract as an eco-friendly and sustainable approach. The synthesized G-CuNPs have shown promising biological activities, including antibacterial, antifungal, and larvicidal effects [27, 58–61].

## 5. Conclusion

CuNPs were successfully biosynthesized using garlic crude extract, providing a low-cost, ecofriendly method with minimal hazardous chemicals. This research demonstrated that G-CuNPs have potent anti-parasitic efficacy against *O. viverrini*, dramatically decreasing parasite motility and resulting in severe tegumental damage, comparable to standard treatments. G-CuNPs also increased ROS levels, indicating their direct anti-parasitic effect. G-CuNPs effectively inhibited the movement of *O. viverrini* adult worms, with no statistically significant differences observed compared to the standard drug treatment or the negative control group. SEM analysis revealed significant damage to the adult worms' tegument, including swelling, desquamating, blistering, and rupturing, with increasing severity at higher G-CuNP concentrations. Currently, our preliminary study revealed that garlic crude extract exhibited moderate cytotoxicity with a half maximal inhibitory concentration (IC50) value of 1.465 mg/mL, as determined through standard cell viability assays. These findings provide foundational insights into its bioactivity, offering a basis for predicting the safety profile of G-CuNPs. However, further research and clinical studies are necessary to elucidate the biological mechanisms of G-CuNPs, evaluate their safety profile through comprehensive cellular studies and animal experiments, and assess their drug delivery capabilities to determine feasibility. Also, the practical applications of G-CuNPs in human treatments should be investigated, focusing on their effectiveness in treating and controlling parasitic infections. Investigating the potential of G-CuNPs as an alternative treatment for *O. viverrini* infections is essential to establish their suitability.

## Figures and Tables

**Table 1:** Garlic crude extract composition obtained from GC-MS analysis. The respective area percentages of identified compounds in the garlic crude extract are presented.

**Retention time**	**Compound names**	**Area summary (%)**
5.069	2-Propenoic acid	0.16
6.970	3-Methyloxirane-2-carboxylic acid	0.23
7.322	Glycolaldehyde dimethyl acetal	0.31
12.648	Limonene	0.05
13.729	Diallyl disulphide	0.41
15.243	Silane, cyclohexyldimethoxymethyl-	0.03
15.976	3-Vinyl-1,2-dithiacyclohex-4-ene	0.19
16.095	Dodecane	0.22
16.473	3-Vinyl-1,2-dithiacyclohex-5-ene	0.43
17.458	Tridecane	0.61
18.030	2-Propenylthioacetonitrile	0.35
19.630	Tetradecane	0.14
20.834	1-Dodecanol	15.21
22.754	Hexadecane	0.10
24.098	Dodecyl acrylate	79.84
28.678	Propanoic acid, 3-mercapto-, dodecyl ester	1.72
**Total**		**100.00**

*Note:* The bold text is used to highlight the listed details and the total value.

**Table 2:** Organic compounds of G-CuNPs. The respective area percentages of the identified compounds in the G-CuNPs are determined.

**Retention time**	**Compound names**	**Area summary (%)**
4.084	Benzoyl isothiocyanate	1.90
6.539	Propane, 1,1-diethoxy-	6.87
10.057	Disulfide, methyl propyl	7.76
12.529	Limonene	0.74
14.288	Disulfide, dipropyl	71.87
22.942	Benzothiazole, 2-(methylthio)-	3.45
24.078	Dodecyl acrylate	3.75
28.309	Hexadecanoic acid, ethyl ester	0.98
40.330	Sulfurous acid, 2-ethylhexyl isohexyl ester	2.68
**Total**		**100.00**

*Note:* The bold text is used to highlight the listed details and the total value.

## Data Availability

This study involved the analysis of new data. The dataset is available from the corresponding author upon reasonable request.
